# Molecular Basis of Growth Inhibition by Acetate of an Adenylate Cyclase-Deficient Mutant of *Corynebacterium glutamicum*

**DOI:** 10.3389/fmicb.2020.00087

**Published:** 2020-02-11

**Authors:** Natalie Wolf, Michael Bussmann, Abigail Koch-Koerfges, Nino Katcharava, Julia Schulte, Tino Polen, Johannes Hartl, Julia A. Vorholt, Meike Baumgart, Michael Bott

**Affiliations:** ^1^IBG-1: Biotechnology, Institute of Bio- and Geosciences, Forschungszentrum Jülich, Jülich, Germany; ^2^Institute of Microbiology, ETH Zürich, Zurich, Switzerland

**Keywords:** *Corynebacterium glutamicum*, cAMP, adenylate cyclase, acetate, uncouplers, membrane potential, GlxR, cytochrome *bc*_1_-*aa*_3_ supercomplex

## Abstract

In *Corynebacterium glutamicum*, cyclic adenosine monophosphate (cAMP) serves as an effector of the global transcriptional regulator GlxR. Synthesis of cAMP is catalyzed by the membrane-bound adenylate cyclase CyaB. In this study, we investigated the consequences of decreased intracellular cAMP levels in a Δ*cyaB* mutant. While no growth defect of the Δ*cyaB* strain was observed on glucose, fructose, sucrose, or gluconate alone, the addition of acetate to these growth media resulted in a severe growth inhibition, which could be reversed by plasmid-based *cyaB* expression or by supplementation of the medium with cAMP. The effect was concentration- and pH-dependent, suggesting a link to the uncoupling activity of acetate. In agreement, the Δ*cyaB* mutant had an increased sensitivity to the protonophore carbonyl cyanide *m*-chlorophenyl hydrazone (CCCP). The increased uncoupler sensitivity correlated with a lowered membrane potential of acetate-grown Δ*cyaB* cells compared to wild-type cells. A reduced membrane potential affects major cellular processes, such as ATP synthesis by F_1_F_*O*_-ATP synthase and numerous transport processes. The impaired membrane potential of the Δ*cyaB* mutant could be due to a decreased expression of the cytochrome *bc*_1_-*aa*_3_ supercomplex, which is the major contributor of proton-motive force in *C. glutamicum*. Expression of the supercomplex genes was previously reported to be activated by GlxR-cAMP. A suppressor mutant of the Δ*cyaB* strain with improved growth on acetate was isolated, which carried a single mutation in the genome leading to an Ala131Thr exchange in GlxR. Introduction of this point mutation into the original Δ*cyaB* mutant restored the growth defect on acetate. This supported the importance of GlxR for the phenotype of the Δ*cyaB* mutant and, more generally, of the cAMP-GlxR system for the control of energy metabolism in *C. glutamicum*.

## Introduction

The Gram-positive soil bacterium *Corynebacterium glutamicum* was identified as a natural glutamate producer in the 1950s (Kinoshita et al., 1957). Since then, various strains of this bacterium are widely used for the production of amino acids, in particular L–glutamate and L–lysine (Eggeling and Bott, 2015). For several years now, *C. glutamicum* has also been employed in commercial protein production (Freudl, 2017). In addition, strains for the synthesis of numerous other industrially relevant compounds have been developed (Schneider and Wendisch, 2011; Becker and Wittmann, 2012; Wieschalka et al., 2013). The success in rational strain development by metabolic engineering is based on detailed studies of the metabolic and regulatory network of *C. glutamicum* (Eggeling and Bott, 2005; Burkovski, 2008; Yukawa and Inui, 2013). Furthermore, efficient novel technologies for strain development involving high-throughput screening approaches with single-cell metabolite biosensors based on transcriptional regulators have been established for *C. glutamicum* (Binder et al., 2012; Mustafi et al., 2012; Schendzielorz et al., 2014; Eggeling et al., 2015).

The transcriptional regulator GlxR, a homolog of the cAMP-receptor protein Crp of *Escherichia coli*, is a global regulator of *C. glutamicum* and activates or represses more than 100 genes. Transcriptional regulation by GlxR influences various cellular functions such as central carbon metabolism, respiration, ATP synthesis, or transport processes (Toyoda et al., 2011; Jungwirth et al., 2013). *In vitro*, purified GlxR binds to DNA when complexed with 3′,5′-cyclic adenosine monophosphate (cAMP) (Kim et al., 2004; Kohl et al., 2008; Bussmann et al., 2009). Crystal structures of apo- and cAMP-bound GlxR were solved and revealed conformational changes of the homodimer upon cAMP binding (Townsend et al., 2014). GlxR showed negative allosteric behavior, as binding of the first cAMP molecule (K_*D*__1_ = 17 μM) reduced the binding affinity of the second cAMP molecule (K_*D*__2_ = 130 μM) to the structurally identical site in the second monomer (Townsend et al., 2014). The affinity of purified GlxR to a double-stranded oligonucleotide containing a central GlxR consensus binding site increased about 100-fold upon cAMP binding from 8.3 μM to 87 nM (Townsend et al., 2014).

The intracellular cAMP level is determined by the rates of synthesis from ATP via adenylate cyclases (Shenoy et al., 2004), degradation to adenosine monophosphate (AMP) via phosphodiesterases (Richter, 2002), and possibly cAMP export and import processes. In *C. glutamicum*, a single adenylate cyclase was identified, which is encoded by the *cyaB* gene (cg0375) (Kalinowski et al., 2003). CyaB contains an N-terminal membrane-integral domain with six predicted transmembrane helices, which is linked via a HAMP domain to a class IIId catalytic domain (CHD). The HAMP domain might function as transmitter domain, as it was found to have a strong positive stimulatory effect on the adenylate cyclase activity of Rv3645 of *Mycobacterium tuberculosis*, which has the same domain composition as CyaB of *C. glutamicum* (Linder et al., 2004). A *C. glutamicum* mutant lacking about 200 bp of the coding region of the catalytic domain of CyaB (strain CgYA) showed strongly reduced cAMP levels both in glucose minimal medium and LB-acetate medium (Cha et al., 2010). Wild-type cAMP levels could be restored by plasmid-encoded *cyaB*, but not by supplementation of cAMP to the medium. Interestingly, this CgYA mutant had a strong growth defect in acetate and glucose-acetate minimal medium, but not in glucose or ethanol minimal medium (Cha et al., 2010). Since the activities of the glyoxylate cycle enzymes isocitrate lyase and malate synthase were even higher in the mutant than in the wild type (WT) during growth on LB-acetate, the authors speculated that the acetate uptake carrier might play a role in the growth defect of the CgYA mutant (Cha et al., 2010). Degradation of cAMP in *C. glutamicum* is catalyzed by the recently identified phosphodiesterase CpdA (Cg2761) (Schulte et al., 2017b). This enzyme belongs to the class II phosphodiesterases and deletion of the *cpdA* gene led to an increase of the intracellular cAMP concentration (Schulte et al., 2017b). The Δ*cpdA* mutant exhibited slower growth and a prolonged lag-phase on all tested carbon sources, including glucose, gluconate, citrate, acetate and ethanol (Schulte et al., 2017b). The growth defects could partially be complemented by overexpression of genes that are normally repressed by the cAMP-GlxR complex, such as *ptsI-ptsG* or *citH*, and that are involved in uptake or metabolism of the respective carbon source. This suggested that mainly the higher fraction of cAMP-bound GlxR caused by the increased cAMP level is responsible for the growth defects of the Δ*cpdA* mutant.

The major aim of our current study was to elucidate the molecular basis of the growth inhibition by acetate of a *C. glutamicum* mutant lacking the *cyaB* gene in order to understand the consequences of a reduced cAMP level. Our results suggest that the inhibitory effect of acetate is caused by its property to act as an uncoupler and that a Δ*cyaB* mutant has a reduced capability of generating membrane potential and possibly ATP by oxidative phosphorylation, which might be due to a reduced transcriptional activation of the genes encoding respiratory chain components and the *atp* operon. Support for the assumption that the growth defect of the Δ*cyaB* strain on acetate is due to a reduced activity of GlxR was obtained by the isolation of a suppressor mutant that had lost the growth defect on acetate. This mutant contained a single amino acid exchange in GlxR. In summary, we show that the cAMP level in combination with the global regulator GlxR plays an important role in the bioenergetics of *C. glutamicum*.

## Materials and Methods

### Strains, Plasmids and Culture Conditions

All strains and plasmids used in this study are listed in Table 1. *C. glutamicum* strains were cultivated either in brain heart infusion (BHI) medium (Bacto^TM^ BHI, BD, Heidelberg, Germany) or in CGXII minimal medium (adjusted to pH 7.0 with KOH) supplemented with 3,4-dihydroxybenzoate (30 mg l^–1^) as iron chelator and different carbon sources (Frunzke et al., 2008) as specified in the results section. For growth experiments, 5 ml BHI medium was inoculated with a single colony and incubated at 30°C and 130 rpm for 8 h. About 400 μl of this first preculture were used for the inoculation of the second preculture, which was cultivated for about 16 h at 30°C and 120 rpm in a 100 ml baffled shake flask containing 20 ml CGXII medium with 2% (w/v) glucose. For the main culture, 800 μl CGXII medium in FlowerPlates (m2p-labs, Baesweiler, Germany) was inoculated to an optical density at 600 nm (OD_600_) of 1 and cultivated in a BioLector microcultivation system (m2p-labs, Baesweiler, Germany) at 1200 rpm, 30°C and 80% humidity. Growth was followed by measuring the backscatter at 620 nm, which reflects the cell density (Kensy et al., 2009). For cultivations in 500 ml shake flasks, 50 ml CGXII medium was inoculated with the second preculture to an OD_600_ of 1. The cultivations in shake flasks were performed at 30°C and 120 rpm and growth was followed by measuring OD_600_. *E. coli* DH5α was used as host for all cloning purposes and was cultivated at 37°C in LB medium (Sambrook and Russell, 2001). When required, media were supplemented with kanamycin (25 μg ml^–1^ for *C. glutamicum* and 50 μg ml^–1^ for *E. coli*).

**TABLE 1:** Bacterial strains and plasmids used in this study.

**Strain or plasmid**	**Relevant characteristics**	**Source or references**
**Strains**		
*Escherichia coli* DH5α	F^–^ *thi-1 endA1 hsdR17*r-m +) *supE44*Δ*lacU169*Φ80*lacZ*Δ*M15*) *recA1 gyrA96 relA1*	Invitrogen
*Escherichia coli* BL21(DE3)	F^–^ *ompT gal dcm lon hsdSB* (rB^–^ mB^–^) λ[DE3 (*lacI lacUV5-T7* gene 1 *ind1 sam7 nin5*)]	Studier and Moffatt, 1986
*Corynebacterium glutamicum* ATCC 13032	ATCC 13032, biotin-auxotrophic wild-type strain (WT)	Kinoshita et al., 1957
*C. glutamicum* Δ*cyaB*	ATCC 13032 with an in frame deletion of the adenylate cyclase gene *cyaB* (cg0375)	This study
*C. glutamicum* Δ*cpdA*	ATCC 13032 with an in frame deletion of the phosphodiesterase gene *cpdA* (cg2761)	Schulte et al., 2017b
*C. glutamicum* Δ*cyaB*Δ*cpdA*	ATCC 13032 with an in frame deletion of the gene *cyaB* and the phosphodiesterase gene *cpdA* (Δ*cyaB*Δ*cpdA*)	This study
*C. glutamicum*Δ*qcr*	ATCC 13032 with an in frame deletion of the *qcrCAB* genes (cg2405, cg2404, cg2403) encoding the three subunits of the cytochrome *bc*_1_ complex	Niebisch and Bott, 2001
*C. glutamicum*Δ*cyaB*_sup1	*C. glutamicum* Δ*cyaB* suppressor mutant carrying a single genomic mutation (C to T) at position 307072 in BA000036.3 leading to the amino acid exchange Ala131Thr in GlxR (cg0350)	This study
*C. glutamicum*Δ*cyaB*_sup2	*C. glutamicum* Δ*cyaB* suppressor mutant carrying two genomic mutations in BA000036.3, one at position 877553 (A to G) located in the intergenic region of *serC* (cg0948) and *gltA* (cg0949) and one at position 1548741 (G to C) located in the intergenic region of *gpt* (cg1659) and cg1660	This study
*C. glutamicum*Δ*cyaB*_sup3	*C. glutamicum* Δ*cyaB* suppressor mutant carrying two genomic mutations in BA000036.3, one at position 307072 (C to T) leading to the amino acid exchange Ala131Thr in GlxR and one at position 2564086 (G to T) leading to a silent mutation of the Gly106 codon of ribose 5-phosphate isomerase (cg2658)	This study
*C. glutamicum::glxR*_A131T	*C. glutamicum* WT in which an Ala131Thr exchange in the *glxR* coding sequence was introduced by double homologous recombination	This study
*C. glutamicum*Δ*cyaB::glxR*_A131T	*C. glutamicum* Δ*cyaB* mutant in which an Ala131Thr exchange in the *glxR* coding sequence was introduced by double homologous recombination	This study
**Plasmids**		
pAN6	Kan^*R*^; P_*tac*_, *lacI*^*q*^ pBL1 oriV_*Cg*_ pUC18 oriV_*Ec*_, *C. glutamicum/E. coli* shuttle vector, derivative of pEKEx2	Frunzke et al., 2008
pAN6-*cyaB*	Kan^*R*^; pAN6 derivative carrying the *cyaB* gene (cg0375) including 300 bp upstream of the start codon and a 3′-terminal StrepTag-II-encoding sequence under the control of an IPTG-inducible *tac* promoter	This study
pAN6-*glxR*-Twinstrep	Kan^*R*^; pAN6 derivative carrying the *glxR* gene (cg0350) including a 3′-terminal Twinstrep-tag encoding sequence before the stop codon (WSHPQFEKGGGSGGGSGGSAWSHPQFEK)	This study
pAN6-*glxR*-A131T-Twinstrep	Kan^*R*^; pAN6-*glxR*-Twinstrep derivative with a Ala131T exchange	This study
pK19*mobsacB*	Kan^*R*^; oriT oriV_*Ec*_ *sacB lacZ*α*;* vector for allelic exchange in *C. glutamicum*	Schäfer et al., 1994
pK19*mobsacB*-Δ*cyaB*	Kan^*R*^; pK19*mobsacB* derivative containing an overlap extension PCR product covering the up- and downstream regions of *cyaB*	This study
pK19*mobsacB*-Δ*cpdA*	Kan^*R*^; pK19*mobsacB* derivative containing an overlap extension PCR product covering the up- and downstream regions of *cpdA*	Schulte et al., 2017b
pK19*mobsacB*-*glxR*_mut	Kan^*R*^; pK19*mobsacB* derivative containing a 800-bp PCR product covering the 3′-terminal 684 bp of *glxR* including the mutation leading to the Ala131Thr exchange and 116 bp of the downstream region	This study

### Construction of Plasmids and Deletion Mutants

Plasmids were constructed by standard cloning procedures (Sambrook and Russell, 2001) using the oligonucleotides listed in Supplementary Table S1. Deletion mutants of *C. glutamicum* were constructed by double homologous recombination as described previously (Niebisch and Bott, 2001). In brief, *C. glutamicum* ATCC 13032 was transformed with the deletion plasmid carrying the up- and downstream regions of the target gene to be deleted. After selection for the first (kanamycin resistance) and second (kanamycin sensitivity, sucrose tolerance) recombination events, Kan^*S*^-Suc^*R*^ clones were analyzed by colony PCR and the PCR product of clones carrying the desired deletion was further verified by sequencing.

For construction of a Δ*cyaB* deletion mutant, it had to be considered that the length of the coding region of *cyaB* varies in different annotations, resulting in proteins of either 347 amino acids (MRPVAA…; Cgl0311 of strain ATCC 13032) (Ikeda and Nakagawa, 2003), 547 amino acids (MDTVLE…; Cg0375 of strain ATCC 13032) (Kalinowski et al., 2003), or 501 amino acids (MKWLWG…; cgR_0397 of strain R) (Yukawa et al., 2007). RNAseq analysis of strain ATCC 13032 identified a single transcriptional start site presumably leading to a leaderless *cyaB* mRNA encoding a protein of 508 amino acids (MSRLLR…) (Pfeifer-Sancar et al., 2013). We therefore assumed the latter size to be the correct one, although additional transcriptional start sites and CyaB variants of other length cannot be excluded. For construction of the Δ*cyaB* mutant, we deleted the entire coding region except for the 5′-terminal 37 codons and the 3′-terminal 12 codons including the stop codon. After the second homologous recombination event, nine kanamycin-sensitive and sucrose-resistant clones were analyzed by colony PCR. Four clones harbored the *cyaB* deletion whereas five clones contained the wild-type fragment. Thus, the Δ*cyaB* deletion mutant was obtained without any difficulties.

### Isolation of Δ*cyaB* Suppressor Mutants With Improved Growth on Acetate

The acetate-sensitive Δ*cyaB* strain was cultivated in CGXII medium with 150 mM potassium acetate as sole carbon source. The culture was prepared as described above. To obtain acetate-tolerant suppressor clones, cultivations were performed for at least 90 h in a BioLector. Cultures that started to grow and reached comparable backscatter values as the WT were streaked out on BHI agar plates and single colonies were inoculated again in CGXII medium with 150 mM potassium acetate. Cultures that grew better than the Δ*cyaB* parental strain were streaked out again on BHI agar plates. The genomic DNA of such clones was isolated and used for whole genome sequencing.

### Genomic DNA Sequencing

DNA of the samples was purified with the DNeasy Blood and Tissue kit (Qiagen, Hilden, Germany) starting with the “pretreatment of Gram-positive bacteria,” as described in the manufacturer’s instructions. The obtained DNA was dried and resuspended in max. 100 μl ddH_2_O. For library preparation, the NEBNext Ultra II DNA Library Prep kit for Illumina (New England Biolabs GmbH, Frankfurt am Main, Germany) was used with 2 μg genomic DNA of each sample following the manufacturer’s instructions. The resulting indexed libraries were quantified using the KAPA Library Quantification kit (VWR International GmbH, Darmstadt, Germany) and normalized for pooling. Sequencing was performed on a MiSeq instrument (Illumina, San Diego, CA, United States) using paired-end sequencing with a read-length of 2 × 150 bp. Data analysis and base calling were accomplished with the CLC Genomics workbench (Qiagen, Hilden, Germany). Reads of the parental Δ*cyaB* strain and the suppressor strains were mapped to the genome sequence BA000036.3 of *C. glutamicum* ATCC 13032 (Ikeda and Nakagawa, 2003).

### Determination of mRNA Levels by Reverse Transcription Quantitative PCR (RT-qPCR)

For quantifying the mRNA levels of *ctaD*, *ctaC*, and *qcrC* in *C. glutamicum* WT and the Δ*cyaB* mutant, RT-qPCR was performed. Cells were grown in CGXII medium containing a glucose-acetate mixture (100 mM each) and harvested at an OD_600_ of 6. Cells were disrupted by the addition of QIAzol Lysis Reagent (Qiagen, Hilden, Germany) followed by bead beating with a Precellys24 device (Peqlab Biotechnologie, Erlangen, Germany). RNA was purified and concentrated with an RNeasy Mini kit (Qiagen, Hilden, Germany) including a DNase I treatment. Reverse transcription of total RNA samples to cDNA was performed using Superscript^TM^ III reverse transcriptase and random primers (Invitrogen, Carlsbad, CA, United States) following the manufacturer’s instructions. For the quantitative PCR, KAPA SYBR^®^ FAST qPCR Master Mix (2×) (Roche, Basel, Switzerland) was used following the manufacturer’s protocol. Primer pairs used for the reactions are listed in Supplementary Table S1. As reference gene, *hpt* (cg2985) was used with the oligonucleotides listed in Supplementary Table S1. Fluorescence measurements and analysis of the results were conducted using a qTower 2.2 and the software qPCR-soft 3.1 (Analytic Jena, Jena, Germany). RNA was isolated from three independent cultures of each strain (biological triplicates) and for each sample technical duplicates were performed.

### Global Gene Expression Analysis Using DNA Microarrays

Preparation of RNA and synthesis of fluorescently labeled cDNA were carried out as described (Möker et al., 2004). Custom-made DNA microarrays for *C. glutamicum* ATCC 13032 printed with 70mer oligonucleotides were obtained from Operon (Cologne, Germany) and are based on the genome sequence entry NC_006958 (Kalinowski et al., 2003). Hybridization and stringent washing of the microarrays were performed according to the instructions of the supplier. Processed and normalized data as well as experimental details (Brazma et al., 2001) were stored in the in-house microarray database for further analysis (Polen and Wendisch, 2004). Using the DNA microarray technology, the genome-wide mRNA concentrations of *C. glutamicum* wild type were compared with those of the mutant strain *C. glutamicum*Δ*cyaB*. The strains were cultivated in CGXII medium with a glucose-acetate mixture (100 mM each). RNA used for the synthesis of labeled cDNA was prepared from cells in the exponential growth phase. Three independent DNA microarray experiments were performed, each starting from independent cultures.

### Determination of cAMP

Cell extracts were prepared as described previously (Schulte et al., 2017b) and the cAMP concentration was measured without dilution with the direct cAMP ELISA kit (Enzo Life Sciences GmbH, Lörrach, Germany) following the manufacturer’s instructions. Amounts of cAMP were related to the protein content of the supernatant of the cell extract. The product specification of the used cAMP ELISA kit (Direct cAMP ELISA kit; Enzo, Lausen, Switzerland) reports the following cross-reactivities with nucleotides other than cAMP, which is set as 100%: AMP, 0.33%; ATP, 0.12%; cyclic GMP, GMP, GTP, cyclic UMP, CTP, all <0.001%. For determining the cross-reactivities, the nucleotides were dissolved in assay buffer to a concentration of 2000 pmol/ml, which is 10-fold higher than the highest concentration used for cAMP in the non-acetylated variant of the assay. The apparent concentrations determined for the non-cAMP nucleotides, e.g., 6.6 pmol/ml for AMP, was divided by the real AMP concentration in the assay (2000 pmol/ml) and multiplied with 100 to give the cross-reactivity in%.

Additionally, intracellular cAMP was measured by LC-MS/MS. *C. glutamicum* cells were grown to the exponential phase (OD_600_ of ∼5). For the sampling, cells from a culture volume corresponding to 4 ml of OD_600_ of 1 were harvested. Sampling, quenching, metabolite extraction and measurements were performed as previously described (Müller et al., 2015; Hartl et al., 2017). Briefly, metabolite separation was achieved by reverse-phase-ion-pairing (tributylamin) liquid chromatography using a nLC-ultra (Eksigent) system. The LC was hyphenated to a QExactive Plus Orbitrap (Thermo Fisher) mass spectrometer with an electrospray ionization probe. The MS was operating in negative mode; the resolution was set to 70,000 (at m/z 200). cAMP was measured using parent reaction monitoring (PRM) with m/z 328.045 as the precursor ion and quantified with the corresponding m/z 134.0465 fragment. Fragmentation was achieved by higher energy collisional dissociations (HCDs) with a normalized collision energy (NCE) of 30.0. cAMP was identified by exact mass (m/z tolerance of 0.003 Da) as well as matching retention time and fragmentation pattern with an analytical 3′,5′-cAMP standard. cAMP was quantified by peak integration using the trapezoid rule; absolute quantification was performed by external calibration. The calibration curve of reference solutions with known cAMP concentrations was fitted by linear regression. The intracellular cAMP concentrations in *Corynebacterium* were estimated assuming a correlation factor of 250 mg cell dry weight (CDW) l^–1^ at OD_600_ of 1 (Kabus et al., 2007) and a corresponding intracellular volume of 1.44 μl per mg CDW (da Luz et al., 2016). The limit of detection (LOD) was estimated to be 14 amol l^–1^ by calculating 3.3 standard deviations of the y-intercepts divided by the slope obtained from linear regression (ICH, 2005) of reference cAMP solutions; the LOD was confirmed by visual inspection. Assuming similar dilution factors as for measured *Corynebacterium* extracts, this corresponds to an LOD of ∼0.1 μmol l^–1^ from cell extracts.

### Measurement of Membrane Potential via Flow Cytometry

The membrane potential of *C. glutamicum* cells was determined by flow cytometry using the fluorescent dye 3,3′-diethyloxacarbocyanine iodide [DiOC2(3)] (Novo et al., 1999, 2000). The assay was performed according to a previously established protocol for *C. glutamicum* (Neumeyer et al., 2013). In brief, the strains were cultivated in baffled shake flasks with 50 ml CGXII medium containing either 100 mM glucose, or 100 mM acetate, or 200 mM acetate (precultures as described for the BioLector cultivation). Measurement of the membrane potential was performed when cells had reached the mid-exponential growth phase (OD_600_ of ∼5). The culture was diluted with FACSFlow^TM^ buffer to an OD_600_ of 0.05 and cells were stained for 30 min with 30 μM DiOC2(3) (3 mM stock solution in dimethyl sulphoxide, Sigma-Aldrich, Germany) and analyzed using a FACS Aria II and BD Diva software (BD Biosciences, Heidelberg, Germany). Green fluorescence was measured at an excitation wavelength of 488 nm and an emission wavelength of 497 nm, red fluorescence at an excitation wavelength of 488 nm and an emission wavelength of 610 nm. For each sample, 100,000 cells were measured at 2000 cells s^–1^. The red/green fluorescence ratio was analyzed using FlowJo V.10 software and plotted as a histogram with GraphPad Prism8.

### Quantitative Determinations of Carbon Sources

Glucose, gluconate and acetate concentrations in culture supernatants were determined as described (Koch-Koerfges et al., 2012) by ion-exchange chromatography using an Agilent 1100 HPLC system (Agilent Technologies, Waldbronn, Germany) equipped with a cation exchange column (Organic Acid Resin 300 × 8 mm, CS Chromatographie Service, Langerwehe, Germany). Isocratic elution within 40 min with 100 mM H_2_SO_4_ and a flow rate of 0.4 ml/min at 40°C was used. Organic acids were detected using a diode array detector at 215 nm and glucose was analyzed by a refraction index (RI) detector in the same run. The quantification of organic acids and glucose was based on a calibration curve with external standards.

### TMPD Enzyme Assay

*N*,*N*,*N*′,*N*′-tetramethyl-*p*-phenylenediamine (TMPD) oxidase activity was measured spectrophotometrically at 562 nm in a 96-well plate with an Infinite M1000 PRO microplate reader (Tecan, Männedorf, Switzerland). TMPD was added to 100 mM Tris–HCl buffer pH 7.5 containing isolated membrane proteins to a final concentration of 200 μM. For the calculation of the TMPD oxidation rate, an extinction coefficient of 10.5 mM^–1^ cm^–1^ was used (Sakamoto et al., 2001). The autoxidation rate of TMPD was recorded using samples containing only buffer and 200 μM TMPD and subtracted from the rates of the membranes. The cells for these measurements were cultivated at 30°C and 90 rpm in 5 l baffled shake flasks with 500 ml CGXII medium and glucose plus acetate (100 mM each) as carbon source. The cultures were harvested in the exponential growth phase at the OD_600_ of 10. The preparation of cell membranes was performed as described (Niebisch and Bott, 2003).

### Electrophoretic Mobility Shift Assays (EMSAs) With Purified GlxR

EMSAs were performed to compare *in vitro* binding of the wild-type GlxR protein (GlxR_WT_) and the variant GlxR_A131T_ to selected DNA target sites. The gene *glxR* was amplified from genomic DNA of *C. glutamicum* WT with the oligonucleotides GlxR-twin1 and GlxR-twin2. A DNA fragment with a Twin-Strep tag-encoding sequence was amplified with the oligonucleotides GlxR-twin3 and GlxR-twin4 using a suitable plasmid with the corresponding sequence as template. Gibson assembly was performed with pAN6 cut with *Nde*I and *Nhe*I, the *glxR* fragment and the Twinstrep-tag fragment resulting in the plasmid pAN6-*glxR*-Twinstrep. The oligonucleotides GlxR-A131T_fw and GlxR-A131T_rv were used to introduce the mutation leading to the amino acid exchange A131T in GlxR using the QuickChange Site-Directed Mutagenesis Kit (Agilent, Waldbronn, Germany). The resulting plasmid was named pAN6-*glxR*-A131T-Twinstrep. Overproduction of GlxR_WT_ or GlxR_A131T_ was performed by cultivation of *E. coli* BL21(DE3) transformed with pAN6-*glxR*-Twinstrep or pAN6-*glxR*-A131T-Twinstrep in ZYM-5052 auto-induction medium (Studier, 2005). After harvesting and disrupting the cells, proteins were purified using Strep-Tactin XT affinity chromatography according to the protocol of the supplier (IBA Life Sciences, Göttingen, Germany). Subsequently, the proteins were further purified by size exclusion chromatography using a Superdex 200 increase column using a buffer composed of 100 mM Tris–HCl pH 7.5, 5% (v/v) glycerol, 100 mM KCl, 20 mM MgCl_2_, and 1 mM EDTA.

EMSAs were performed as described previously (Bussmann et al., 2009) using the following DNA fragments: (i) a 140 bp DNA fragment upstream of *ctaD* extending from −127 to −261 upstream of the *ctaD* start codon; (ii) a 132 bp DNA fragment upstream of *ctaCF* extending from −102 to −234 upstream of the *ctaC* start codon; and (iii) a 136 bp DNA fragment covering an intragenic region of the gene cg3153. The first two DNA fragments contain previously described GlxR binding sites (Kohl et al., 2008; Toyoda et al., 2011). The respective DNA fragments were generated by PCR and purified with the DNA Clean & Concentrator Kit (Zymo Research, Freiburg im Breisgau, Germany).

## Results and Discussion

### cAMP Levels in *C. glutamicum* WT and Mutants Lacking *cyaB* or *cpdA*

In order to confirm previous studies with the *C. glutamicum* mutant strain CgYA lacking approximately 200 bp coding region of the catalytic domain of the adenylate cyclase CyaB (Cha et al., 2010), we constructed another Δ*cyaB* mutant lacking almost the entire coding region (see section “Materials and Methods”). The cAMP level of this mutant was compared with that of the WT and the Δ*cpdA* mutant lacking the cAMP phosphodiesterase (Schulte et al., 2017b) using an enzyme-linked immunosorbent assay (ELISA). The strains were grown in CGXII glucose medium to an OD_600_ of about 5 (exponential growth phase). As shown in Table 2, the cAMP level of the Δ*cyaB* mutant (∼20 pmol (mg protein)^–1^) amounts to only 20% of the cAMP level of the WT (∼100 pmol (mg protein) ^–1^), whereas that of the Δ*cpdA* mutant was higher (∼144 pmol (mg protein) ^–1^). These results confirmed that the lack of the adenylate cyclase CyaB causes a strong decrease of the cAMP level, which is in agreement with previous results (Cha et al., 2010; Toyoda et al., 2011; Schulte et al., 2017b). It should be noted that the absolute values obtained in the ELISA measurements published in these studies differ significantly.

**TABLE 2:** cAMP levels in various *C. glutamicum* strains cultivated in CGXII medium with 100 mM glucose.

***C. glutamicum* strain**	**Intracellular cAMP concentration^1^**	
	**pmol mg^–1^ protein (determined by ELISA)**	**μM (determined by LC-MS/MS)**
WT	99.9 ± 31.8	0.9 ± 0.4
Δ*cyaB*	20.5 ± 4	n.d.^2^
Δ*cpdA*	144.1 ± 7.9	3.4 ± 1.5
Δ*cyaB*Δ*cpdA*	30 ± 6.8	n.d.

*^1^Each value represents the mean value with the standard deviation of three biological replicates. ^2^n.d., not detectable.*

Bioinformatic analysis revealed only a single adenylate cyclase-encoding gene (*cyaB*) in the genome of *C. glutamicum*. Therefore, deletion of *cyaB* should result in the complete lack of the cAMP. However, both in our Δ*cyaB* mutant as well as in previously analyzed *cyaB* mutants of strain ATCC 13032 (Cha et al., 2010) and strain R (Toyoda et al., 2011), a residual cAMP level was measured by ELISA assays, raising the question for the source of this residual cAMP. The kit used in this study (Enzo Life Sciences GmbH, Lörrach, Germany) shows low cross-reactivity to AMP and ATP, which might contribute to the residual signal in the Δ*cyaB* mutant. Alternatively or additionally, *C. glutamicum* might possess another enzyme with adenylate cyclase activity besides CyaB, which contributes to the residual cAMP level in the Δ*cyaB* mutants and which is not detectable in bioinformatic searches using members of the known classes of adenylate cyclases (Shenoy et al., 2004). If a yet unknown novel enzyme with adenylate cyclase activity exists in *C. glutamicum*, a deletion of the *cpdA* gene in the Δ*cyaB* background might lead to an increase of cAMP. Therefore, we constructed a Δ*cyaB*Δ*cpdA* double mutant and determined the cAMP level by the ELISA kit. Although a small increase was detected for the double mutant, the difference to the Δ*cyaB* mutant was not significant (Table 2). In growth experiments with glucose or acetate minimal medium, the Δ*cyaB*Δ*cpdA* mutant behaved like the Δ*cyaB* mutant (Supplementary Figure S1), arguing against the existence of an alternative adenylate cyclase besides CyaB.

As the ELISA-based assay might be influenced by cross-reacting metabolites, LC-MS/MS was applied as an alternative method to quantify cAMP levels. With this method, we determined an absolute concentration of cAMP of ∼1 μM in wild-type cells, whereas a ∼3.5-fold higher concentration was determined in the Δ*cpdA* mutant. cAMP was not detected in the Δ*cyaB* mutant and in the Δ*cyaB*Δ*cpdA* double mutant. These results support the possibility that the residual cAMP levels determined by the ELISA assay in the latter two mutants might be caused by cross-reactivities and suggest that CyaB is the only adenylate cyclase present in *C. glutamicum*.

### Sensitivity of the Δ*cyaB* Mutant to Acetate

**FIGURE 1:**
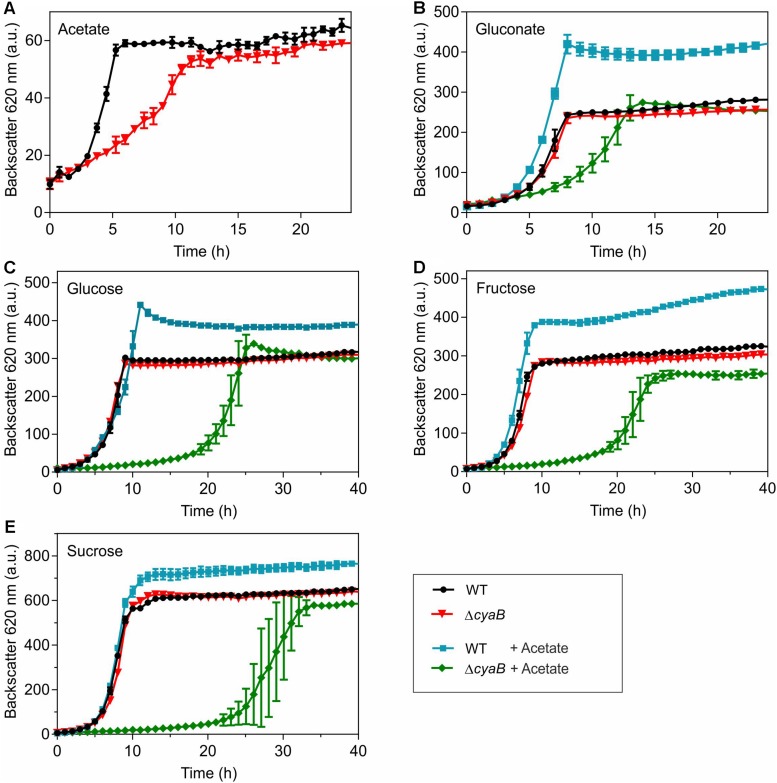
Growth of *C. glutamicum* WT and the Δ*cyaB* mutant in CGXII medium with **(A)** 100 mM acetate, **(B)** 100 mM gluconate or gluconate-acetate mixture (100 mM each), **(C)** 100 mM glucose or glucose-acetate mixture (100 mM each), **(D)** 100 mM fructose or fructose-acetate mixture (100 mM each), and **(E)** 100 mM sucrose or sucrose-acetate mixture (100 mM each). Mean values and standard deviations of three biological replicates are shown.

Growth of the Δ*cyaB* mutant and the WT with different carbon sources was compared using the BioLector cultivation system. In CGXII minimal medium containing 100 mM of either glucose, gluconate, fructose, or sucrose as carbon source, the Δ*cyaB* mutant grew like the WT, whereas a clear growth defect was observed for the mutant when 100 mM acetate served as carbon source (Figure 1). In media containing mixtures of gluconate and acetate, glucose and acetate, fructose and acetate, or sucrose and acetate (100 mM of each carbon source), the Δ*cyaB* mutant showed a strong growth defect, whereas the WT was unaffected and grew to higher cell densities (measured as higher backscatter values) (Figure 1). The data obtained with the Δ*cyaB* mutant for glucose, acetate, and the glucose-acetate mixture are in agreement with those described previously for the CgYA mutant, whereas the results for fructose-acetate and sucrose-acetate disagree, as growth of the CgYA mutant on these mixtures was reported not to be impaired (Cha et al., 2010). Our results indicate that acetate inhibits growth of the Δ*cyaB* mutant irrespective of the presence of an additional carbon source.

With respect to the inhibitory effect of acetate, ethanol is a particularly interesting carbon source, as its catabolism in *C. glutamicum* involves acetate as an intermediate. Ethanol degradation proceeds via the initial oxidation to acetaldehyde by alcohol dehydrogenase followed by a second oxidation to acetate by acetaldehyde dehydrogenase (Arndt and Eikmanns, 2007; Arndt et al., 2008). Acetate is then converted via acetyl phosphate to acetyl-CoA by acetate kinase and phosphotransacetylase and acetyl-CoA is oxidized in the TCA cycle with the glyoxylate cycle serving as an anaplerotic reaction (Wendisch et al., 2000; Gerstmeir et al., 2003; Bott, 2007). As shown in Supplementary Figure S2, the Δ*cyaB* mutant grew like the WT in CGXII medium containing 150 mM ethanol as carbon source. This result is in agreement with data reported previously for the CgYA mutant (Cha et al., 2010) and shows that acetate degradation is functional in the Δ*cyaB* mutant.

**FIGURE 2:**
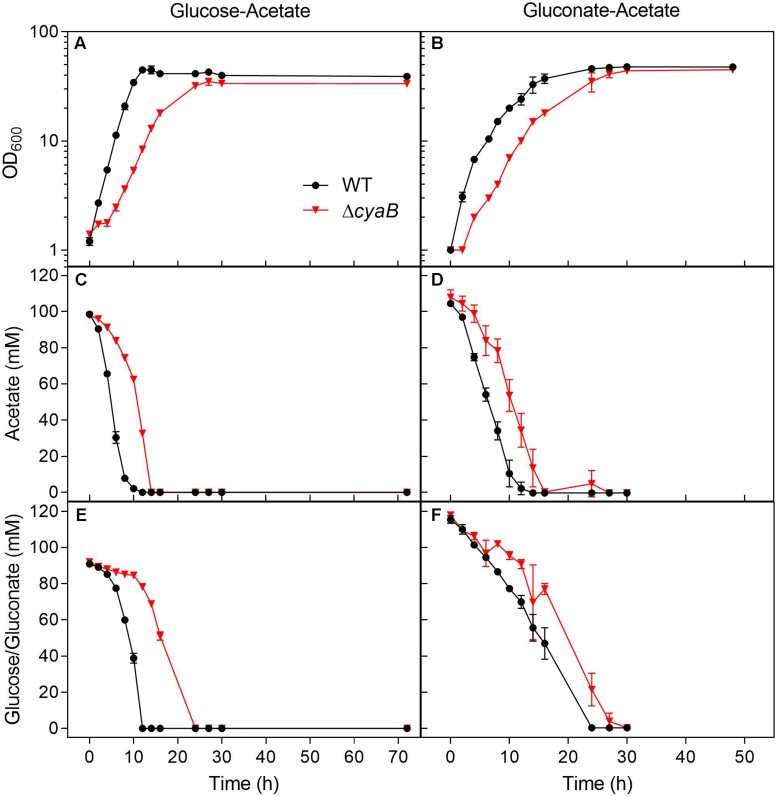
Growth and substrate consumption of *C. glutamicum* WT and its Δ*cyaB* mutant in CGXII minimal medium containing either **(A)** glucose and acetate (100 mM each) or **(B)** gluconate and acetate (100 mM each). Panels **(C,D)** show the acetate consumption by the cultures displayed in panels **(A,B)**. Panel **(E)** shows the glucose consumption of the cultures displayed in panel **(A)**. Panel **(F)** shows the gluconate consumption of the cultures displayed in panel **(B)**. The strains were cultivated at 30°C and 120 rpm in 500 ml baffled shake flasks containing 50 ml CGXII minimal medium with the depicted carbon source. Mean values and standard deviations of three biological replicates are shown.

Growth of the WT and the Δ*cyaB* mutant was also compared in shake flasks in order to be able to follow the consumption of the carbon sources during cultivation (Figure 2). The growth defect of the Δ*cyaB* mutant during growth on a glucose-acetate mixture (100 mM each) was confirmed and resulted in a retarded consumption of both carbon sources compared to the WT. In CGXII medium containing a gluconate-acetate mixture, the Δ*cyaB* mutant also showed a growth defect and retarded consumption of gluconate and acetate compared to the WT. In contrast to glucose, fructose, and sucrose, gluconate is not taken up via the PEP-dependent phosphotransferase system (PTS), but via the secondary transporter GntP (Frunzke et al., 2008). The fact that acetate was completely consumed confirms that acetate catabolism is functional in the Δ*cyaB* mutant.

### Abolishment of the Growth Defect of the Δ*cyaB* Mutant in the Presence of Acetate

**FIGURE 3:**
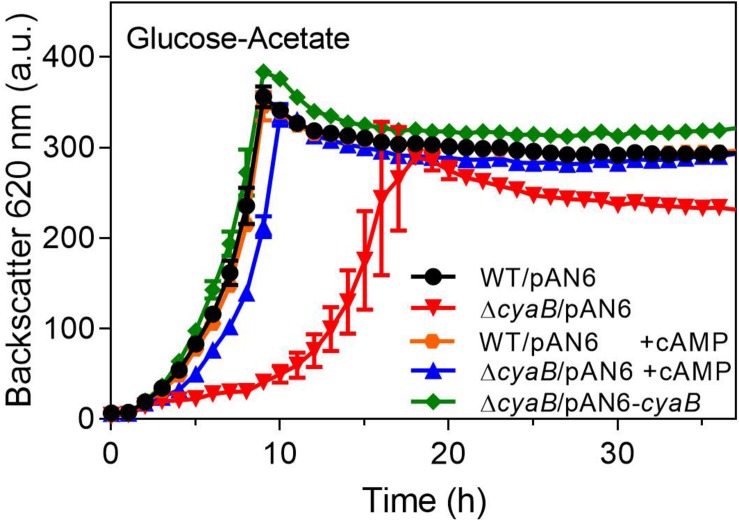
Complementation of the Δ*cyaB* mutant with plasmid-encoded *cyaB* (pAN6-*cyaB*) or the addition of 10 mM extracellular cAMP (+ cAMP). The cultures contained 0.25 mM IPTG, 25 μg ml**^–^**^1^ kanamycin and, where indicated, 10 mM cAMP. Mean values and standard deviations of three biological replicates are shown.

The Δ*cyaB* mutant and the WT were transformed with the *cyaB* expression plasmid pAN6-*cyaB* or the parent vector pAN6. As shown in Figure 3, the growth defect of the Δ*cyaB* mutant in glucose-acetate medium was abolished by plasmid-based expression of *cyaB*, but not by the presence of the vector alone. This result confirmed that the *cyaB* deletion rather than a hypothetical secondary mutation that might have occurred during mutant construction was responsible for the acetate sensitivity. In a previous study, we showed that cAMP addition to the medium influences GlxR-based gene expression, indicating that it can enter the cell (Schulte et al., 2017a). We therefore tested the influence of cAMP addition on growth of the Δ*cyaB* mutant and could show that 10 mM cAMP abrogated the growth defect in the presence of acetate, confirming that it is due to a lowered cAMP level (Figure 3). In the case of the CgYA mutant, addition of cAMP to the medium could not reverse the growth inhibition (Cha et al., 2010). The reason for this discrepancy is unknown. Uptake of 3′,5′-cAMP was previously reported for *E. coli* and marine bacteria, but no distinct transporter could be identified (Saier et al., 1975; Goldenbaum and Hall, 1979; Ammerman and Azam, 1982).

### Concentration and pH Dependency of Growth Inhibition of the Δ*cyaB* Mutant by Acetate

As described above, the inhibitory effect of acetate on growth of the Δ*cyaB* mutant was independent of the presence of additional carbon sources. The difference when comparing growth on ethanol and growth on acetate is that during ethanol degradation acetate is only a metabolic intermediate that does not accumulate to high concentrations but is directly catabolized. In contrast, when acetate is used as carbon source, it is present in a high concentration outside of the cell. It was speculated that inhibition of acetate uptake by the secondary monocarboxylate transporter MctC (Cg0953) might play a role for the growth defect (Cha et al., 2010). However, under the conditions used in our studies (100 mM acetate, pH 7), uptake of acetate by passive diffusion is completely sufficient, as shown by the fact that growth of a *mctC* deletion mutant on acetate at pH 7 was unaffected (Jolkver et al., 2009).

**FIGURE 4:**
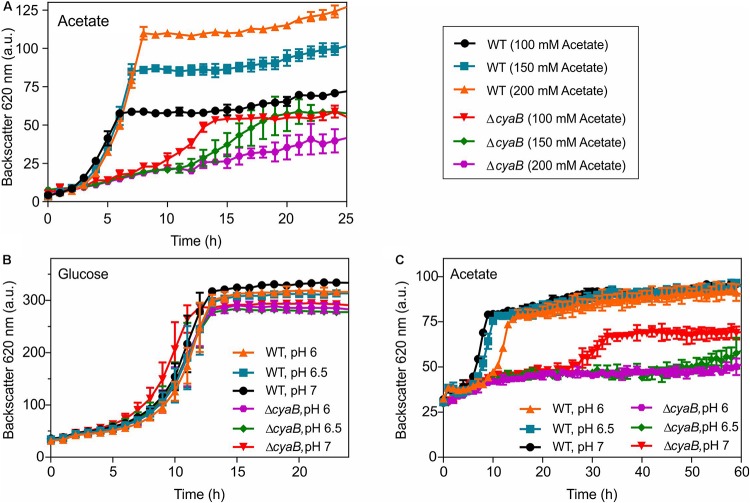
Growth of *C. glutamicum* WT and the Δ*cyaB* mutant in CGXII medium with increasing sodium acetate concentrations **(A)**. pH dependency of growth of *C. glutamicum* WT and the Δ*cyaB* mutant in modified CGXII medium containing 20 g l^–1^ MOPS and 20 g l^–1^ MES buffer (pH adjusted with either KOH or HCl) with either 100 mM glucose **(B)** or 100 mM acetate **(C)** as carbon source. Mean values and standard deviations of three biological replicates are shown.

Based on the above considerations, we assumed that the inhibitory effect of acetate on growth of the Δ*cyaB* mutant is due to its property to act as an uncoupler (Axe and Bailey, 1995; Pinhal et al., 2019). We tested whether the inhibitory effect of acetate is concentration- and pH-dependent. Weak acids such as acetate become more effective as proton translocator when the pH gets closer to their p*K*_*a*_ (4.76 in the case of acetic acid). In a first set of experiments, the WT and the Δ*cyaB* mutant were grown in CGXII medium containing 100 mM, 150 mM, or 200 mM acetate. In the case of the WT, increased acetate concentrations led to increased cell densities, but did not affect the growth rate strongly. In contrast, growth inhibition of the Δ*cyaB* mutant clearly correlated with increasing acetate concentrations (Figure 4A). The dose-dependent negative impact of acetate on growth of the Δ*cyaB* mutant was also observed in cultivations in CGXII medium with 100 mM glucose and either 50 mM or 100 mM acetate (Supplementary Figure S3). Furthermore, the inhibitory effect of acetate was observed independent of whether sodium acetate or potassium acetate were used for preparation of the media, showing that this effect is not caused by the cation (data not shown). For testing the pH dependency of growth inhibition by acetate, the MOPS buffer of the standard CGXII medium was substituted by a mixture of 20 g l^–1^ MOPS (p*K*_*a*_ 7.20) and 20 g l^–1^ MES (p*K*_*a*_ 6.15) and pH values of 6.0, 6.5, and 7.0 were adjusted by addition of KOH or HCl. Besides media with 100 mM acetate, also media with 100 mM glucose were used at the indicated pH values. As shown in Figure 4B, growth of the WT and the Δ*cyaB* mutant in glucose medium was comparable and hardly affected by the initial pH value. In contrast, growth of the mutant in acetate medium was strongly affected by the pH, showing almost no growth at pH 6.5 and pH 6.0 (Figure 4C). In summary, the inhibitory effect of acetate on growth of the Δ*cyaB* mutant was both concentration- and pH-dependent, supporting our assumption that it is due to the uncoupling properties of acetate.

### Uncoupler Sensitivity and Membrane Potential of WT and the Δ*cyaB* Mutant

**FIGURE 5:**
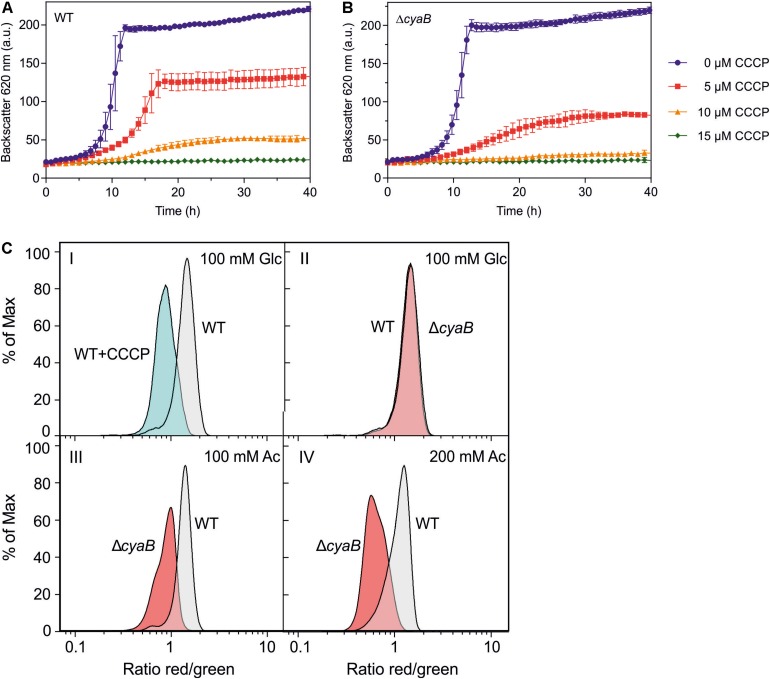
Growth of *C. glutamicum* WT **(A)** and the Δ*cyaB* mutant **(B)** in CGXII medium with 2% (w/v) glucose in the presence of different CCCP concentrations. Mean values and standard deviations of three biological replicates are shown. **(C)** Relative comparison of the membrane potential (ΔΨ) of *C. glutamicum* cells by flow cytometry of [DiOC2(3)]-stained cells. Overlays of the histograms obtained with WT cells and Δ*cyaB* mutant cells are shown. The percentage of analyzed cells (% of Max) is plotted versus the logarithm of the red/green fluorescence ratio. This ratio serves as an indicator of ΔΨ, with a high value corresponding to a high ΔΨ. Panel I shows a control experiment with glucose-grown WT cells that were treated for 15 min without or with 50 μM CCCP to collapse ΔΨ. Panels II, III, and IV show comparisons of WT and Δ*cyaB* cells cultivated in CGXII medium with either 100 mM glucose (II), 100 mM acetate (III), or 200 mM acetate (IV). Representative histograms of three biological and three technical replicates each are shown. The histograms were generated with the software FlowJo V.10 and processed in GraphPad Prism8.

The results described above suggested that the Δ*cyaB* mutant is more sensitive to uncouplers than the WT. To further confirm this assumption, the influence of the protonophore carbonyl cyanide *m*-chlorophenyl hydrazone (CCCP) (Heytler and Prichard, 1962) on growth in CGXII medium with glucose was tested (Figures 5A,B). The Δ*cyaB* mutant was more sensitive to CCCP than the WT. In the presence of 5 μM CCCP, the mutant was stronger inhibited than the WT. In the presence of 10 μM CCCP, the mutant was not able to grow any more, while the WT showed residual growth. At 15 μM CCCP, neither strain was able to grow.

The increased sensitivity to acetate and CCCP of the Δ*cyaB* mutant compared to the WT might be due to a reduced capability of the mutant to build up pmf, which is composed of the membrane potential Δψ and the pH gradient ΔpH (Nicholls and Ferguson, 2002). At pH 7, the pmf of *C. glutamicum* WT is about 200 mV and formed almost exclusively by the membrane potential of 180 mV (Follmann et al., 2009; Koch-Koerfges et al., 2012). We compared Δψ of the WT and the Δ*cyaB* mutant using the dye 3,3′-diethyloxacarbocyanine iodide [DiOC_2_(3)], which exhibits green fluorescence in all stained bacteria and shifts toward red emission due to self-association of dye molecules in dependency of Δψ. Higher Δψ correlates with increased red fluorescence and the ratio of red/green fluorescence gives a relative measure of the membrane potential (Novo et al., 1999, 2000). This method does not give absolute values for the membrane potential but is useful for relative comparison of the membrane potential of different strains.

Using a previously established protocol (Neumeyer et al., 2013) we first confirmed that treatment of wild-type cells grown in CGXII glucose medium with 50 μM CCCP led to the expected decrease of the red/green fluorescence ratio indicating a collapsed or strongly reduced Δψ (Figure 5C, panel I). When cells of the WT and the Δ*cyaB* mutant cultivated in glucose medium to the exponential growth phase were analyzed, no significant differences of the red/green ratio was observed, indicating that both strains had a comparably high Δψ (Figure 5C, panel II). The histograms differed, however, when the strains were cultivated in CGXII medium with 100 mM acetate as carbon source. In this case, the mean fluorescence ratio of wild-type cells was almost unaltered, whereas that of the Δ*cyaB* mutant was strongly decreased (Figure 5C, panel III). This shift of the red/green ratio indicates that the membrane potential of the Δ*cyaB* mutant is reduced compared to the membrane potential of the WT. For wild-type cells cultivated with 200 mM acetate, the mean fluorescence ratio was reduced compared to cells grown with glucose or 100 mM acetate, in line with the concentration-dependent uncoupling effect of acetate, but the ratio of the Δ*cyaB* mutant was much stronger affected (Figure 5C, panel IV). These results indicate that acetate affects Δψ of the Δ*cyaB* mutant much more strongly than Δψ of the WT.

### Role of the Cytochrome *bc*_1_-*aa*_3_ Supercomplex for Acetate Sensitivity

The results described above suggest that the cAMP deficiency of the Δ*cyaB* mutant causes a reduced ability to maintain a high membrane potential in the presence of the uncoupler acetate. In *C. glutamicum* cultivated under oxic conditions, pmf is generated by the cytochrome *bc*_1_-*aa*_3_ supercomplex (6 H^+^/2 e^–^) and by cytochrome *bd* oxidase (2 H^+^/2 e^–^) (Bott and Niebisch, 2003; Niebisch and Bott, 2003; Kabashima et al., 2009). We previously showed that Δψ is reduced in a Δ*qcr* mutant lacking a functional supercomplex, whereas it is comparable to the WT in a Δ*cydAB* mutant lacking cytochrome *bd* oxidase (Koch-Koerfges et al., 2013). Therefore, a link between the cAMP level and the activity of the respiratory supercomplex might exist.

The transcriptional regulator GlxR is the only protein currently known in *C. glutamicum* whose activity is controlled by the cAMP level. In the presence of cAMP, purified GlxR was shown to bind to double-stranded 40-mer oligonucleotides covering predicted GlxR-binding sites in the promoter regions of the *ctaCF* operon, the *ctaE-qcrCAB* operon, and the *ctaD* gene, which encode the subunits of the cytochrome *bc*_1_-*aa*_3_ supercomplex (Kohl et al., 2008). In glucose-grown cells of strain R, chromatin affinity chromatography followed by DNA chip analysis of the enriched DNA fragments (ChIP-chip) confirmed that Strep-tagged GlxR binds *in vivo* to these proposed binding sites (Toyoda et al., 2011). Mutation of the GlxR-binding sites in the *ctaC* and *ctaD* promoter regions of genomically integrated single-copy transcriptional fusions reduced expression of the reporter gene *lacZ* in yeast extract-containing medium with either glucose or acetate by about 15 – 40% (Toyoda et al., 2011). These results indicated that GlxR acts as a transcriptional activator of the genes encoding the cytochrome *bc*_1_-*aa*_3_ supercomplex. The reduced cAMP level in the Δ*cyaB* mutant might thus cause a reduced expression of the genes for the *bc*_1_-*aa*_3_ supercomplex leading to a reduced capacity to build up membrane potential and to counteract the uncoupling activity of acetate. A transcriptome comparison of the Δ*cyaB* mutant with the WT revealed reduced expression of all genes encoding the supercomplex (Supplementary Table S2). Additionally, we also performed qRT-PCR of *ctaC*, *ctaD*, and *qcrC* and the resulting data also showed decreased expression of these genes in the Δ*cyaB* mutant (Supplementary Table S2).

To test for differences of cytochrome *aa*_3_ oxidase activity in the Δ*cyaB* mutant and the WT, the TMPD oxidase activity of membrane fractions of the two strains grown in CGXII medium with glucose-acetate was determined. TMPD is supposed to donate electrons to cytochrome *c*_1_. The TMPD oxidase activity of the Δ*cyaB* mutant was 35% reduced (280 ± 11 nmol TMPD oxidized min^–1^ (mg membrane protein) ^–1^) compared to the WT (433 ± 22 nmol TMPD oxidized min^–1^ (mg membrane protein) ^–1^), supporting the assumption that a reduced activity of the *bc*_1_-*aa*_3_ supercomplex contributes to the uncoupler sensitivity of the mutant during growth on acetate.

**FIGURE 6:**
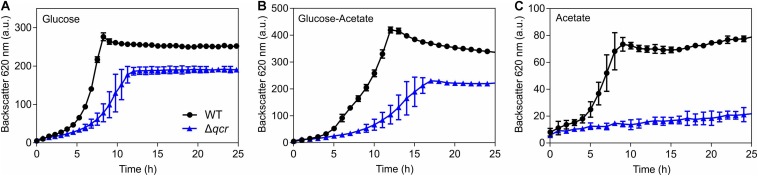
Growth of the indicated *C. glutamicum* strains in CGXII medium with either 100 mM glucose **(A)**, 100 mM glucose plus 150 mM potassium acetate **(B)**, or 150 mM potassium acetate as carbon source **(C)**. Mean values and standard deviations of three biological replicates are shown.

To further support the relevance of the cytochrome *bc*_1_-*aa*_3_ supercomplex for the acetate sensitivity of the Δ*cyaB* mutant, growth of the WT and the Δ*qcr* mutant was compared in CGXII medium with glucose, glucose plus acetate, or acetate (Figure 6). Already in glucose medium the Δ*qcr* mutant showed a growth defect, as known from previous studies (Niebisch and Bott, 2001). In medium with glucose and acetate, the growth defect of the Δ*qcr* mutant became more severe, whereas in minimal medium with acetate as sole carbon source the Δ*qcr* mutant showed no growth. These results support the assumption that the cytochrome *bc*_1_-*aa*_3_ branch of the respiratory chain is crucial for growth on acetate.

Growth on acetate does not allow net ATP synthesis by substrate level phosphorylation and is strictly dependent on oxidative phosphorylation by F_1_F_O_-ATP synthase (Koch-Koerfges et al., 2012). Interestingly, also the expression of the *atpBEFHAGDC* operon (cg1362-cg1369) encoding the eight subunits of F_1_F_O_-ATP synthase was shown to be activated by GlxR (Toyoda et al., 2011) and therefore might be lowered in the Δ*cyaB* mutant. The transcriptome comparison supported this assumption with about two-fold lowered mRNA levels of the *atp* genes in the Δ*cyaB* mutant. Consequently, the growth defect of the mutant on acetate is most likely additionally caused by a reduced ATP synthesis via oxidative phosphorylation, caused by reduced synthesis of the *bc*_1_-*aa*_3_ supercomplex and F_1_F_O_-ATP synthase.

### Isolation of Suppressor Mutants of the Δ*cyaB* Strain With Improved Growth on Acetate

To get further insights into the molecular basis of the acetate sensitivity of the Δ*cyaB* mutant, we isolated Δ*cyaB* suppressor mutants that show improved growth on acetate. As shown in Supplementary Figure S4A, three independent cultures started to grow after about 80 h of incubation in acetate minimal medium. After plating on BHI agar plates, single colonies were picked from each of the three cultures and tested again for growth on acetate. As shown in Supplementary Figure S4B, two of the suppressor mutants, named Δ*cyaB*_sup1 and Δ*cyaB*_sup3, grew almost like the WT, whereas the third one, Δ*cyaB*_sup2, showed slower growth than the other two mutants. Genomic DNA of the three suppressor mutants was isolated and sequenced with average coverages of 82, 81, and 106 for Δ*cyaB* _sup1, Δ*cyaB* _sup2, and Δ*cyaB* _sup3, respectively. For strain Δ*cyaB*_sup1, a single point mutation was identified compared to the parent Δ*cyaB* mutant at position 307072 (numbering according to BA000036.3), which is located in the *glxR* gene and leads to an Ala131Thr exchange. The frequency of the mutation in the Δ*cyaB*_sup1 mutant was 100%. Interestingly, the same mutation was also identified in strain Δ*cyaB*_sup3, which additionally carried a second point mutation at position 2564086 (frequency 97.0%) leading to a silent mutation in codon 106 (Gly) of the ribose 5-phosphate isomerase gene *rpi* (cg2658). The suppressor mutant Δ*cyaB*_sup2 did not carry a mutation in *glxR*, but two point mutations in intergenic regions, one between promoters P1 and P2 of *gltA* (cg0949; citrate synthase) (van Ooyen et al., 2011) at position 877553 (A to G, frequency 100%) and the other one 39 bp upstream of the start codon of cg1660, encoding a putative manganese efflux pump (position 1548741, G to C, frequency 100%). The position of the point mutation in the *gltA* promoter does not overlap with known regulator binding sites for RamA and GlxR (van Ooyen et al., 2011) and therefore the effect of this mutation cannot be predicted. Similarly, the effect of the second mutation upstream of cg1660 cannot be deduced as neither the promoter nor the regulation of this gene is known.

The finding that two of the three independently obtained suppressor mutants carried the same mutation (A131T) in GlxR supports the crucial role of this regulator for the acetate sensitivity of the Δ*cyaB* mutant. To confirm that this mutation can rescue the acetate sensitivity of the Δ*cyaB* strain, it was introduced by site-directed mutagenesis into the genome of the Δ*cyaB* mutant and for comparison into the WT. The resulting strains Δ*cyaB*::*glxR*_A131T and WT::*glxR*_A131T showed comparable growth in minimal medium with glucose compared to the parental strains Δ*cyaB* and the WT (Supplementary Figure S4C). Importantly, the Δ*cyaB*::*glxR*_A131T strain showed wild-type like growth in acetate minimal medium, confirming the phenotype of strain Δ*cyaB* _sup1 (Supplementary Figure S4D).

The alanine residue at position 131 of GlxR is highly conserved in homologs from other *Corynebacterium* species and *Mycobacterium tuberculosis* as well as in CRP of *E. coli* (Supplementary Figure S4E). Ala131 is located in the central α-helix of GlxR, which forms the dimer interface, and is positioned close to important cAMP-binding residues (Townsend et al., 2014) (Supplementary Figures S4E,G). Overlays of the crystal structures of apo- and holo-GlxR and of models with the Ala131T exchange are shown in Supplementary Figures S4F,G. The mutation apparently does not lead to large structural changes, making it difficult to predict the functional consequences of the amino acid exchange. Due to the vicinity of residue 131 to the cAMP-binding site, the A131T exchange might have altered the influence of cAMP on DNA-binding. We therefore tested whether purified GlxR-A131T still requires cAMP for binding to DNA targets in electrophoretic mobility shift assays. As shown in Supplementary Figure S5, binding of GlxR-A131T to DNA fragments covering the GlxR-binding sites in front of *ctaD* and *ctaC* was still dependent on cAMP. However, the *in vivo* situation is probably different. ChIP-Chip experiments with a *cyaB*-deletion strain of *C. glutamicum* R clearly showed GlxR-binding to target sites despite the lack of CyaB, although with decreased affinity (Toyoda et al., 2011).

The most intensively studied homolog of GlxR is CRP of *E. coli*. Many studies were performed in which *E. coli*Δ*cya* strains were used to select for mutants that regained the ability to grow on carbon sources such as lactose, maltose, or xylose requiring activation of gene expression by CRP. These studies have recently been summarized (Frendorf et al., 2019) and led the authors to conclude that adaptive mutations occur predominantly in the cAMP binding site, the D-α-helix, and in the RNA polymerase activating domains AR1 and AR2, which likely affect ligand binding, ligand-induced allosteric transitions, or the productive interaction with the core RNA polymerase, respectively. In these studies, no mutation was found in CRP residue A121, which corresponds to A131 of GlxR. However, it has to be considered that although the 3-dimensional fold of GlxR and CRP is very similar, the structural changes observed for GlxR upon cAMP binding are quite distinct from those observed for CRP (Townsend et al., 2014). It was concluded that the mechanisms of allosteric binding and activation of DNA-binding differ considerably in the CRP/FNR family without dramatic structural changes and that the same 3-dimensional fold is finetuned using small structural changes coupled with changes in dynamic behavior to achieve the optimal combination of allostery and DNA recognition (Townsend et al., 2014). This situation makes a prediction of the effects of the A131T mutation in GlxR based on studies of *E. coli* CRP virtually impossible.

## Conclusion

The aim of this study was to elucidate the consequences of a reduced cAMP level in *C. glutamicum* caused by the lack of the adenylate cyclase CyaB and in particular the inhibitory effect of acetate on growth of the Δ*cyaB* mutant. Our results strongly suggest that this effect is mainly caused by the uncoupling activity of acetate, as it is concentration- and pH-dependent and occurs also in the presence of an additional carbon source such as glucose, fructose, sucrose, or gluconate. Evidence was obtained that the Δ*cyaB* mutant has a lower Δψ on acetate than the WT, suggesting a reduced capability to build up pmf. As the growth defect of the Δ*cyaB* mutant could be rescued by supplementation of the medium with cAMP, a link between the major second messenger cAMP and the ability to pmf generation was proposed. The major contributor to pmf in *C. glutamicum* is the cytochrome *bc*_1_-*aa*_3_ supercomplex and there is evidence from previous studies that expression of the genes encoding the supercomplex is activated by the cAMP-dependent transcriptional regulator GlxR. We observed reduced expression of the supercomplex genes and a reduced TMPD oxidase activity in the Δ*cyaB* mutant, supporting the idea that a decreased supercomplex activity contributes to the acetate sensitivity of the Δ*cyaB* mutant. Also the F_1_F_O_-ATP synthase genes are known to be transcriptionally activated by GlxR and showed reduced expression in the Δ*cyaB* mutant, additionally contributing to the energetic deficiencies of the strain. We could rescue the growth defect of the Δ*cyaB* mutant on acetate by a single point mutation (A131T) in GlxR, confirming the key role of GlxR for the phenotype of the Δ*cyaB* mutant. Additional studies are required to elucidate the functional consequences of this amino acid exchange *in vivo*. In summary, our results disclosed that cAMP in concert with GlxR plays a key role in the control of energy metabolism in *C. glutamicum*.

## Data Availability Statement

This manuscript contains previously unpublished data. The DNA microarray data are available in the GEO database with accession number GSE140408. The genome sequencing data (bam files) are available in the ENA database via accession number PRJEB36438.

## Author Contributions

NW and MBu constructed mutants and plasmids and performed all experimental work except the one specified below for other authors. AK-K performed the analysis of glucose and organic acids. NK and JS performed the growth experiments with the protonophore CCCP and the determination of the membrane potential. TP supervised the genome resequencing and analyzed the resulting data. JH and JV performed the LC-MS/MS measurements for cAMP determination. MBa coached the experimental work and supported the design of the study. All authors contributed to the interpretation of the data. NW wrote the first draft of the manuscript and prepared the figures and tables. MBo designed the study, supervised the experimental work, and wrote the final version of the manuscript.

## Conflict of Interest

The authors declare that the research was conducted in the absence of any commercial or financial relationships that could be construed as a potential conflict of interest.
