# Supplementary material for: Molecular Basis of Growth Inhibition by Acetate of an Adenylate Cyclase-Deficient Mutant of Corynebacterium glutamicum
Source: Front Microbiol. 2020 Feb 11;11:87. doi: 10.3389/fmicb.2020.00087 (PMC7026483; doi:10.3389/fmicb.2020.00087)
Supplement: Supplementary file 1 [file Data_Sheet_1.pdf]

Supplementary Material

1 Supplementary Tables

TABLE S1. Oligonucleotides used in this study

Oligonucleotide	Sequence (5’ → 3’) and properties[1]
**Construction of deletion plasmid pK19*mobsacB-cyaB* and PCR analysis of the resulting mutants**	
del0375_1_*Hind*III-fw	ATTAAAGCTTCGGGGTGGCTGCCTCCCATG
del0375_2-rv	CGTTTAGGTTTAGTGGCTGGGCAAACAGCATTACTGCGAGCGCACC
del0375_3-fw	CCCAGCCACTAAACCTCCCCGGCGGCCTATTCGGCCGACGTTG
del0375_4_*Xba*I-rv	ATTATCTAGACAATCACGCCGCGTACATCGC
cg0375_deltest-fw	CAATTGCTGCGGGACGATGTG
cg0375_deltest-rv	GATTCACTGCCTAAAGGTGCG
**PCR analysis of *C. glutamicum* Δ*cyaB*Δ*cpdA***	
cg2761_deltest-fw	GCACAGTGGGAACCATTAAC
cg2761_deltest-rv	GTGGTCGTGATTGTACTTCC
**Construction of plasmid pAN6-*cyaB* and sequencing of the insert**	
pAN6-*cyaB*-Pst-fw	AAAACTGCAGATAGACAATTGCTGCGGG
pAN6-*cyaB*-NheI-rv	AAAAGCTAGCGGACCTATCCGCCAACGTC
pAN6_seq_fw	TTACGCCAAGCTTGCATG
pAN6_seq_rv	GTAAAACGACGGCCAGTG
**Construction of WT::*glxR*_A131T and Δ*cyaB*::*glxR*_A131T mutant and PCR analysis of the mutation**	
cg0350_mut_A131T_fw	CCTGCAGGTCGACTCTAGAGTTCGTTACCTGCAGGCTCAGGAAGCTTC
cg0350_mut_A131T_rv	GTAAAACGACGGCCAGTGAATTGTGGAAGGTGTACAGGAGATCCTGTC
cg0350_PCR_fw	TGACCGCCTTTACATCATCACCTC
cg0350_PCR_rv	CCTGGTCATACCCGTGATTCTGTGTC
cg0350_mut_control	TCGAGCGCGACGTGCCAAATGC
**RT-qPCR analysis**	
RT-*ctaD*-fw1	TGAACAGCTGGTTGAACTGC
RT-*ctaD*-rv1	TCCTTCAGCTTCTTCTTCCTCG
RT-*ctaC*-fw1	GCATTACCCCTGAAGCAGTG
RT-*ctaC*-rv1	ATGGCGGTGAGGAATAGACC
RT-*qcrC*-fw1	CTGCCACAACTTCACTGGTC
RT-*qcrC*-rv1	TAGGCATGTTCTGAGGACCG
RT_*hpt*_fw	ATGTTCCAGCCAACCCATAC
RT_*hpt*_rv	TCTTCGGCGTCTTTGAACTC
**Construction of plasmids pAN6-*glxR*-Twinstrep and pAN6-*glxR*_A131T-Twinstrep**	
GlxR-twin1	GCCTGCAGAAGGAGATATACAGTGGAAGGTGTACAGGAG
GlxR-twin2	TCGAGCGCGACGTGCCAAATGC
GlxR-twin3	GGCACGTCGCGCTCGATGGAGTCATCCTCAATTCG
GlxR-twin4	AAACGACGGCCAGTGAATTTTATTTTTCGAACTGCGGGTG
GlxR-A131T_fw	TCCTGCGCGTTCTGACTCGTCGTCTGCGTCGC
GlxR-A131T_rv	GCGACGCAGACGACGAGTCAGAACGCGCAGGA
**DNA fragments for EMSAs**	
EMSA_*ctaC*_fw	GGTGGAAATATCTTCGTGGGTTTCG
EMSA_*ctaC*_rv	GTTGATGGTCTGTGACGTGG
EMSA_*ctaD*_fw	CTGTATCCCCTTTTCATGC
EMSA_*ctaD*_rv	CTTCCTGGCGAAATGTCCG
EMSA_neg _fw	AGCTGCTGCGTTCAGGTGTC
EMSA_neg_rv	TAGTGGCGGTGGATCAGG

[1]Restriction sites are underlined

TABLE S2. mRNA ratios (Δ*cyaB*/WT) of the genes encoding F_1F_O-ATP synthase and the cytochrome bc_1-aa_3 supercomplex.[1]

Locus tag	Gene	Function	mRNA ratio Δ*cyaB*/WT	
			DNA microarrays[2]	qRT-PCR[3]
cg1362	*atpB*	ATP synthase subunit A	0.51	n.d.[4]
cg1363	*atpE*	ATP synthase subunit C	0.51[5]	n.d.
cg1364	*atpF*	ATP synthase subunit B	0.53	n.d.
cg1365	*atpH*	ATP synthase subunit δ	0.55	n.d.
cg1366	*atpA*	ATP synthase subunit α	0.41	n.d.
cg1367	*atpG*	ATP synthase subunit γ	0.40	n.d.
cg1368	*atpD*	ATP synthase subunit β	0.43	n.d.
cg1369	*atpC*	ATP synthase subunit ε	0.83	n.d.
cg2406	*ctaE*	cytochrome aa_3 oxidase subunit 3	0.67	n.d.
cg2409	*ctaC*	cytochrome aa_3 oxidase subunit 2	0.63	0.20
cg2408	*ctaF*	cytochrome aa_3 oxidase subunit 4	0.31	n.d.
cg2780	*ctaD*	cytochrome aa_3 oxidase subunit 1	0.69	0.32
cg2405	*qcrC*	cytochrome c_1	0.74	0.30
cg2404	*qcrA*	Rieske iron-sulfur protein	0.64	n.d.
cg2403	*qcrB*	cytochrome *b*	0.64	n.d.

[1]Cells of the Δ*cyaB* mutant and the WT were grown in CGXII medium with glucose plus acetate (100 mM each).
[2]mRNA ratios represent mean values of at least two DNA microarray analyses starting from independent cultures.
[3]qRT-PCR results represent mean values of three biological replicates and two technical replicates each
[4]n.d., not determined
[5]except for cg1363, the p-values for all other genes were ≤ 0.05

2 Supplementary Figures

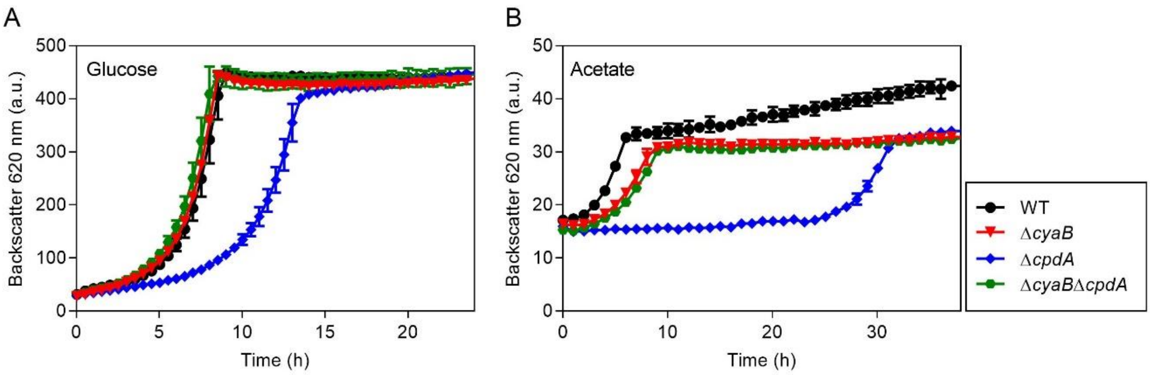

FIGURE S1. Growth of *C. glutamicum* WT and the mutant strains Δ*cyaB*, Δ*cpdA*, and Δ*cyaB*Δ*cpdA* in CGXII medium with 2% (w/v) glucose (**A**) or 100 mM sodium acetate (**B**). Mean values and standard deviations of three biological replicates are shown.

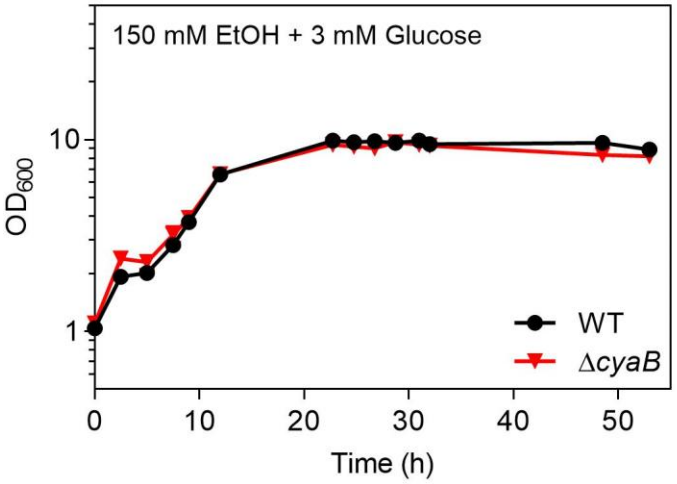

FIGURE S2. Growth of *C. glutamicum* WT and the Δ*cyaB* mutant in CGXII minimal medium with 150 mM ethanol and 3 mM glucose. Cultivation was performed in baffled shake flasks that were incubated at 30 °C and 120 rpm at 85% humidity. Mean values and standard deviations of three biological replicates are shown.

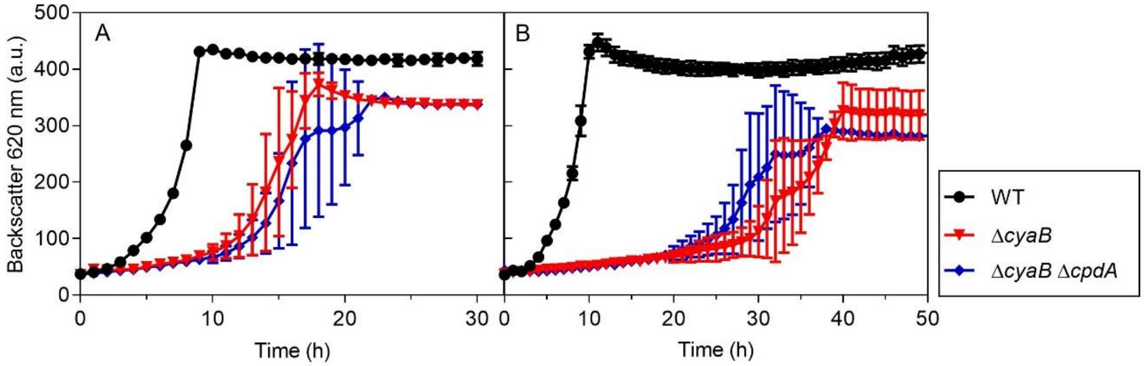

FIGURE S3. Growth of *C. glutamicum* WT and mutant strains Δ*cyaB* and Δ*cyaB*Δ*cpdA* in CGXII medium with 100 mM glucose and 50 mM potassium acetate (**A**) or with 100 mM glucose and 100 mM potassium acetate (**B**). Mean values and standard deviations of three biological replicates are shown.

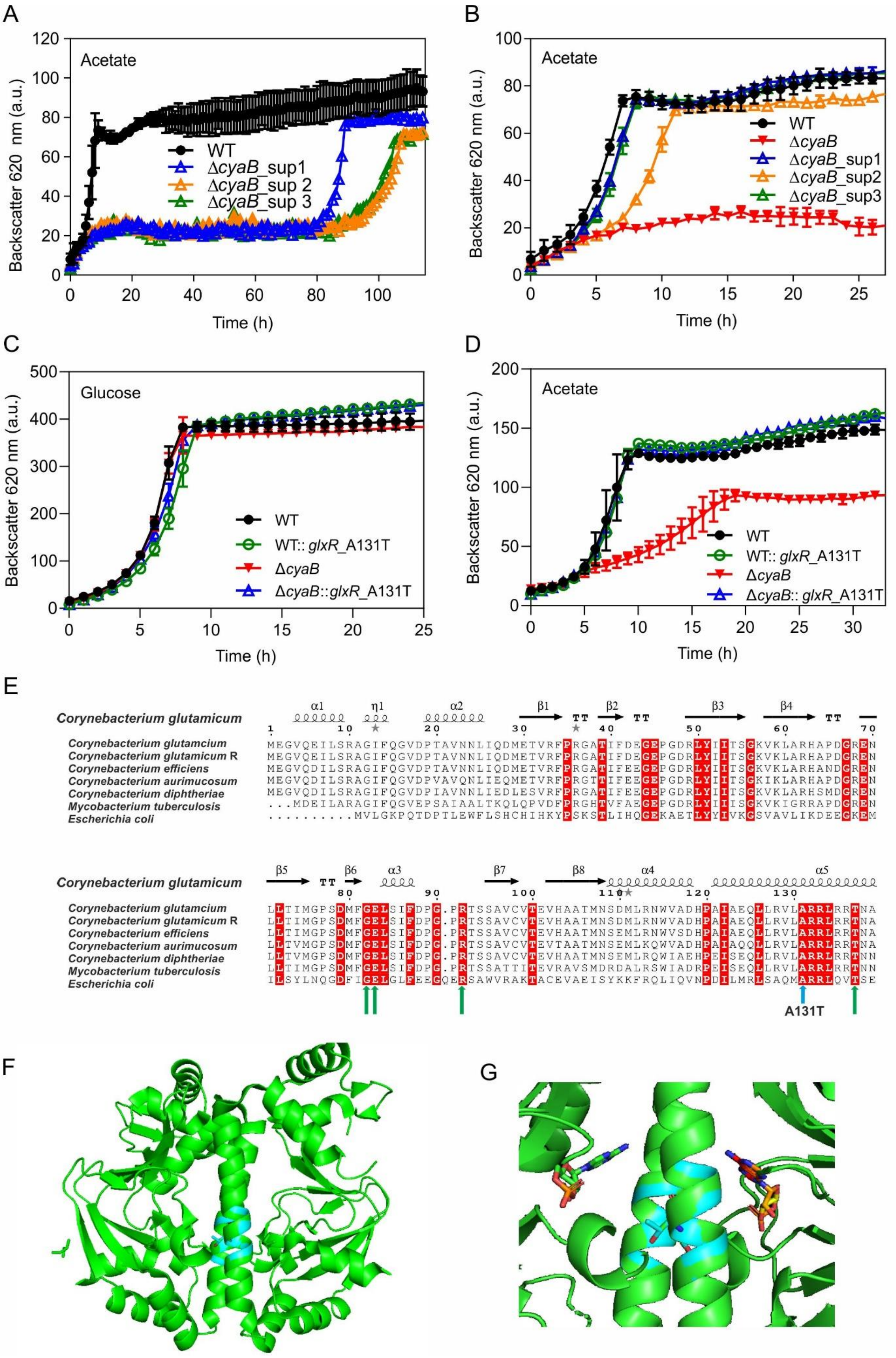
A
Acetate
Backscatter 620 nm (a.u.)
Time (h)
WT
ΔcyaB_sup1
ΔcyaB_sup 2
ΔcyaB_sup 3
B
Acetate
Backscatter 620 nm (a.u.)
Time (h)
WT
ΔcyaB
ΔcyaB_sup1
ΔcyaB_sup2
ΔcyaB_sup3
C
Glucose
Backscatter 620 nm (a.u.)
Time (h)
WT
WT:: glxR_A131T
ΔcyaB
ΔcyaB::glxR_A131T
D
Acetate
Backscatter 620 nm (a.u.)
Time (h)
WT
WT:: glxR_A131T
ΔcyaB
ΔcyaB:: glxR_A131T
E
Corynebacterium glutamicum
Corynebacterium glutamcium MEGVQEILSRAGIFQGVDPTAVNNLIQDMETVRFPRGATIFDEGEPGDRLYIITSGKVKLARHAPDGREN
Corynebacterium glutamicum R MEGVQEILSRAGIFQGVDPTAVNNLIQDMETVRFPRGATIFDEGEPGDRLYIITSGKVKLARHAPDGREN
Corynebacterium efficiens MEGVQDILSRAGIFQGVDPTAVNNLIQDMETVRFPRGATIFEEGEPGDRLYIITSGKVKLARHANDGREN
Corynebacterium aurimucosum MEGVQDILSRAGIFQGVDPVAVQNLIEQMETVRFPRGTTIFEEGEPGDRLYIITSGKIKLARHAPDGREN
Corynebacterium diphtheriae MEGVQDILSRAGIFQGVDPIAVNNLIEDLESVRFPRGATIFEEGEPGDRLYIITSGKVKLARHSMDGREN
Mycobacterium tuberculosis ...MDEILARAGIFQGVEPSAIAALTKQLQPVDFPRGHTVFAEGEPGDRLYIIISGKVKIGRRAPDGREN
Escherichia coli ..........MVLGKPQTDPTLEWFLSHCHIHKYPSKSTLIHQGEKAETLYYIVKGSVAVLIKDEEGKEM
Corynebacterium glutamicum
Corynebacterium glutamcium LLTIMGPSDMFGELSIFDPG.PRTSSAVCVTEVHAATMNSDMLRNWVADHPAIAEQLLRVLARRLRRTNA
Corynebacterium glutamicum R LLTIMGPSDMFGELSIFDPG.PRTSSAVCVTEVHAATMNSDMLRNWVADHPAIAEQLLRVLARRLRRTNA
Corynebacterium efficiens LLTIMGPSDMFGELSIFDPG.PRTSSAVCVTEVHAATMNSEMLRNWVSDHPAIAEQLLRVLARRLRRTNA
Corynebacterium aurimucosum LLTVMGPSDMFGELSIFDPG.PRTSSAVCVTEVTAATMNSEMLKQWVADHPAIAQQLLRVLARRLRRTNA
Corynebacterium diphtheriae LLTVMGPSDMFGELSIFDPG.PRTSSAVCVTEVHAATMNSEMLRQWIAEHPEISEQLLRVLARRLRRTNN
Mycobacterium tuberculosis LLTIMGPSDMFGELSIFDPG.PRTSSATTITEVRAVSMDRDALRSWIADRPEISEQLLRVLARRLRRTNN
Escherichia coli ILSYLNQGDFIGELGLFEEGQERSAWVRAKTACEVAEISYKKFRQLIQVNPDILMRLSAQMARRLQVTSE
A131T
F
G

FIGURE S4. (A) Generation of suppressor mutants of *C. glutamicum* Δ*cyaB* with restored growth on acetate in a long-term cultivation in CGXII medium with 150 mM potassium acetate. **(B)** Growth of single colonies of the Δ*cyaB* suppressor mutant in CGXII medium with 150 mM acetate using *C. glutamicum* WT and the Δ*cyaB* mutant as controls. **(C, D)** Growth of indicated strains in CGXII medium with 100 mM glucose or 100 mM acetate. The *glxR*_A131T mutation identified in the Δ*cyaB* suppressor mutant was introduced by homologous recombination into the genomes of the WT and the Δ*cyaB* mutant. **(E)** Section of an amino acid sequence alignment of GlxR homologs from different bacterial strains. GlxR (Cg0350) of *C. glutamicum* WT and features derived from its crystal structure (PDB 4CYD) were used as reference. Red boxed amino acids are conserved in all selected sequences. Green arrows indicate residues important for cAMP binding in GlxR according to structural analysis (Townsend et al., 2014). The blue arrow shows the position of the A131T exchange in the Δ*cyaB*_sup1 mutant. The alignment was performed with Clustal W (https://www.genome.jp/tools-bin/clustalw) and processed with ESPript 3 (Robert and Gouet, 2014). The following sequences were used for the alignment: *C. glutamicum* strain R CgR0377; *Corynebacterium efficiens* YS-314 CE0287; C*orynebacterium aurimucosum* ATCC700975 Cauri0205; *Corynebacterium diphtheriae* NCTTC13129 CDIP0303; *Mycobacterium tuberculosis* H37Rv Rv3676; *Escherichia coli* MG1655 b3357. (**F**) Overlay of apo-GlxR$_{WT}$ (green) (PDB:4BYY) and model of apo-GlxR$_{A131T}$ (light blue). (**G**) Overlay of holo-GlxR$_{WT}$ (green) (PDB:4CYD) and a model of holo-GlxR$_{A131T}$ (light blue) showing an enlargement of the region close to A131T amino acid exchange including bound cAMP. The model of GlxR-A131T and the overlay were generated with PyMOL.

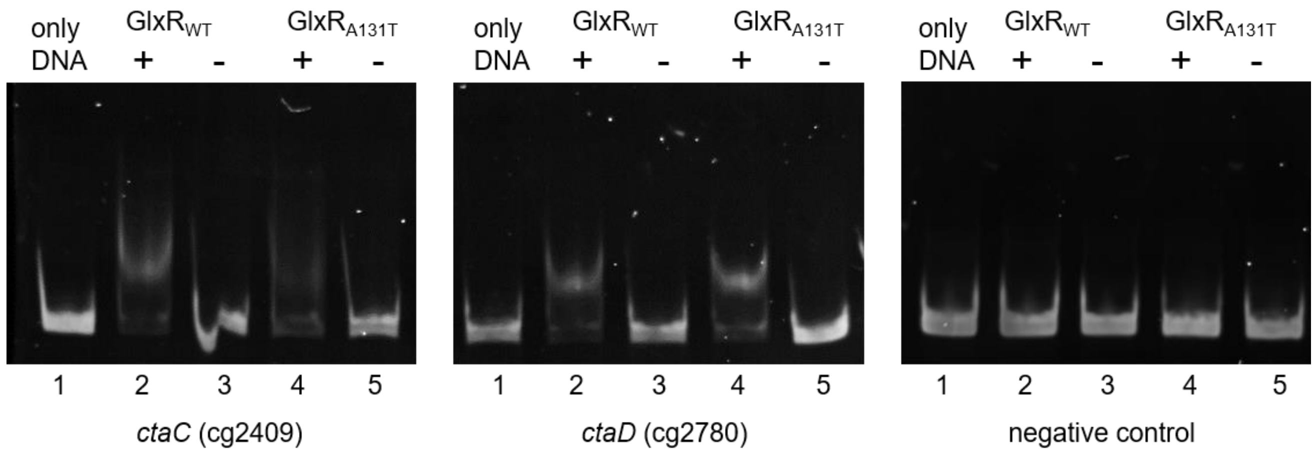

FIGURE S5. Electrophoretic mobility shift assays (EMSAs) with C-terminally Twin-Streptagged GlxR$_{WT}$ or GlxR$_{A131T}$ and DNA fragments covering the known GlxR-binding sites in the promoter regions of *ctaD* (-121 to -261 bp upstream of *ctaD* start codon) and *ctaCF* (-102 to -234 bp upstream of *ctaC* start codon) (Kohl et al., 2008; Toyoda et al., 2011) and an intragenic DNA fragment of cg3153 serving as negative control. 100 ng of the DNA fragments were incubated for 30 min at room temperature with 200 nM purified protein, either with (+) or without (-) 0.2 mM cAMP. After incubation, the reaction mixture was loaded on a 10% native polyacrylamide gel. Lane 1: control sample containing only DNA; lane 2: sample with GlxR$_{WT}$, the indicated DNA fragment, and cAMP; lane 3: sample with GlxR$_{WT}$ and the indicated DNA fragment, but without cAMP; lane 4: sample of GlxR$_{A131T}$ with the indicated DNA fragment and cAMP; lane 5: sample with GlxR$_{A131T}$ and the indicated DNA fragment, but without cAMP.

3 Supplementary References

Kohl, T.A., Baumbach, J., Jungwirth, B., Pühler, A., and Tauch, A. (2008). The GlxR regulon of the amino acid producer *Corynebacterium glutamicum*: *In silico* and *in vitro* detection of DNA binding sites of a global transcription regulator. *J. Biotechnol.* 135(4)**,** 340-350. doi: 10.1016/j.jbiotec.2008.05.011.

Robert, X., and Gouet, P. (2014). Deciphering key features in protein structures with the new ENDscript server. *Nucleic Acids Res.* 42**,** W320-324. doi: 10.1093/nar/gku316.

Townsend, P.D., Jungwirth, B., Pojer, F., Bussmann, M., Money, V.A., Cole, S.T., et al. (2014). The crystal structures of apo and cAMP-bound GlxR from *Corynebacterium glutamicum* reveal structural and dynamic changes upon cAMP binding in CRP/FNR family transcription factors. *PLoS One* 9(12)**,** e113265. doi: 10.1371/journal.pone.0113265.

Toyoda, K., Teramoto, H., Inui, M., and Yukawa, H. (2011). Genome-wide identification of *in vivo* binding sites of GlxR, a cyclic AMP receptor protein-type regulator in *Corynebacterium glutamicum*. *J. Bacteriol.* 193**,** 4123-4133. doi: 10.1128/JB.00384-11.
